# The first crop plant genetically engineered to release an insect pheromone for defence

**DOI:** 10.1038/srep11183

**Published:** 2015-06-25

**Authors:** Toby J.A. Bruce, Gudbjorg I. Aradottir, Lesley E. Smart, Janet L. Martin, John C. Caulfield, Angela Doherty, Caroline A. Sparks, Christine M. Woodcock, Michael A. Birkett, Johnathan A. Napier, Huw D. Jones, John A. Pickett

**Affiliations:** 1 Rothamsted Research, Harpenden, AL5 2JQ, UK

## Abstract

Insect pheromones offer potential for managing pests of crop plants. Volatility and instability are problems for deployment in agriculture but could be solved by expressing genes for the biosynthesis of pheromones in the crop plants. This has now been achieved by genetically engineering a hexaploid variety of wheat to release (*E*)-β-farnesene (*E*βf), the alarm pheromone for many pest aphids, using a synthetic gene based on a sequence from peppermint with a plastid targeting amino acid sequence, with or without a gene for biosynthesis of the precursor farnesyl diphosphate. Pure *E*βf was produced in stably transformed wheat lines with no other detectable phenotype but requiring targeting of the gene produced to the plastid. In laboratory behavioural assays, three species of cereal aphids were repelled and foraging was increased for a parasitic natural enemy. Although these studies show considerable potential for aphid control, field trials employing the single and double constructs showed no reduction in aphids or increase in parasitism. Insect numbers were low and climatic conditions erratic suggesting the need for further trials or a closer imitation, in the plant, of alarm pheromone release.

Insect pheromones, such as the aphid alarm pheromone and other types of behaviour modifying semiochemicals, have long been considered as potential alternatives to current use of broad spectrum eradicant pesticides having toxic modes of action. Although formulations have been designed to accommodate intrinsic high volatility and instability, use in broad-acre agriculture remains limited and costly, aggravated by problems of chemical synthesis and purity. However, production and release from crop plants could solve these problems and create a sustainable pest management approach not requiring seasonal application and its associated carbon footprint. Biotechnological production of moth pheromones by transient expression of synthetic genes in plants has recently been demonstrated^1^.

For many species of pest aphids (Homoptera: Aphididae), the alarm pheromone comprises the sesquiterpene hydrocarbon (*E*)-β-farnesene (*E*βf)^2^, which causes dispersal and repellency, and also increases foraging by natural enemies of aphids such as parasitic wasps (Hymenoptera: Braconidae), and predators^3^,^4^,^5^. Furthermore, *E*βf has been shown to be used by the wild potato, *Solanum berthaultii* (Solanaceae), in defence against aphids^6^, but in this case release is from specialised foliar trichomes allowing it to be produced away from sites of biosynthesis of other sesquiterpenes, which can inhibit the alarm activity of *E*βf^7^. Modification of a gene related to an *E*βf synthase from the peppermint, *Mentha piperita*^8^ (Lamiaceae), enabled the model plant *Arabidopsis thaliana* (Brassicaceae) to be genetically engineered to produce *E*βf sufficiently pure to effect high activity in laboratory studies against the pest aphid *Myzus persicae* and increase foraging behaviour by the parasitoid *Diaeretiella rapae,* both of these species being able to exploit brassicaceous ecosystems^3^. The scene was thereby set to explore this approach in a crop plant, to enable testing both in the laboratory and in the field.

## Results

The hexaploid wheat, *Triticum aestivum* cv. Cadenza (Poaceae), which can be spring or autumn sown, was genetically engineered to express the enzymes (*E*)-β-farnesene synthase (*E*βfS) and the precursor farnesyl diphosphate synthase (FPPS). A total of 62 transgenic events with Eβ*fS* or Eβ*fS* plus *FPPS* were analysed. Expression of the genes was confirmed by semi-quantitative RT-PCR, but no *E*βf could be detected by high resolution gas chromatography (GC) analysis or coupled GC-mass spectrometry (GC-MS) even with single ion monitoring for the diagnostic ion m/z 204 (Fig. S1). Furthermore, no behavioural activity to entrainment volatiles, different from those of untransformed Cadenza, was detected. These data are in contrast to those previously observed by us in Arabidopsis, and represent an interesting but unexpected divergence of sesquiterpene biosynthesis in Angiosperms.

**Figure 1:**
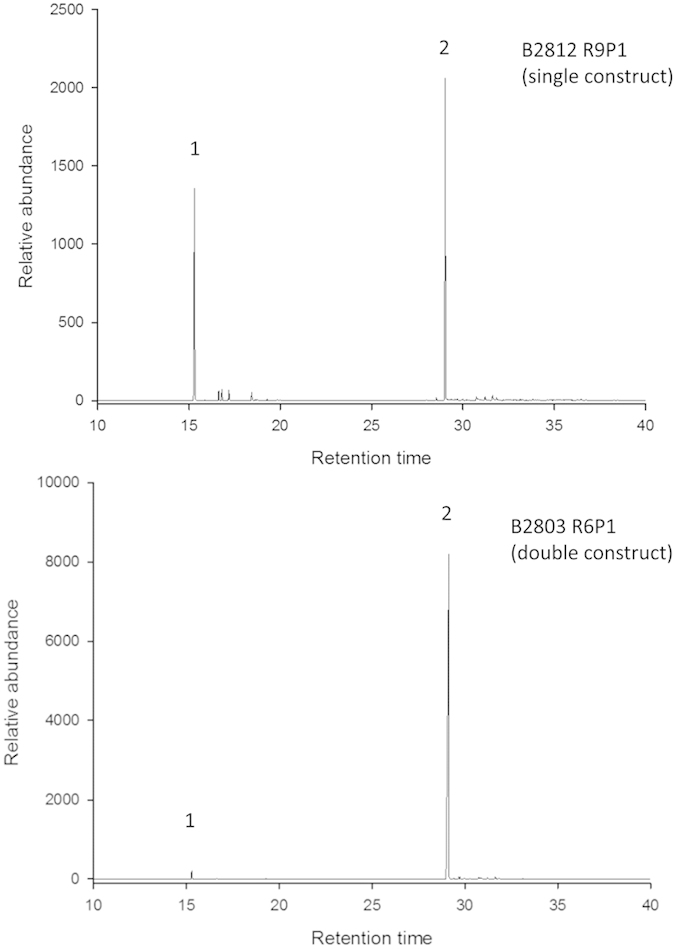
Gas chromatography (GC) analysis of volatiles released from transformed wheat plants 71 days after sowing: (upper trace) B2812 R9P1 possessing the Eβ*fS* gene alone, (lower trace) B28203 R6P1 possessing Eβ*fS* and *FPPS* , both with plastidial targeting sequence,. 1 = myrcene, 2 = (*E*)- β-farnesene (*E*βf). Note different scales on y-axis.

There is evidence that targeting terpene synthases to plastids (via additional *N*-terminal subcellular targeting determinants) can increase levels of terpene biosynthesis^9^,^10^. The next step was to include a transit sequence from the small subunit of RuBisCo, previously validated to target correctly the proteins to wheat plastids^11^,^12^. Wheat plants expressing plastidially-targeted forms of the two constructs, both *E*βfS alone (47 events) and *E*βfS plus FPPS (34 events), were found to generate *E*βf (Fig. 1). From these, we chose two lead events with which to conduct lab- and field-based insect behaviour studies. One (B2803 R6P1) possessed both Eβ*fS* and *FPPS* and displayed a high level of *E*βf emission and one (B2812 R9P1) that possesses Eβ*fS* alone and displayed *E*βf emission in the mid-range of our observations. All the plants that emitted *E*βf had an otherwise normal phenotype, were fully fertile and did **not** display any obvious evidence of somaclonal mutations.

### Copy number and zygosity analysis

A combination of *E*βf emission levels, transgene copy number and inheritance ratios was used to screen the 81 events possessing plastidially targeted *E*βfS or *E*βfS plus FPPS and two lead events were selected (B2803 R6P1 and B2812 R9P1), (Table S1). TaqMan PCR on genomic DNA revealed that event B2803 R6P1 possessed one copy of the Eβ*fS* gene and *FPPS* gene per haploid genome and event B2812 R9P1 possessed four copies of the Eβ*fS* gene per haploid genome. TaqMan analysis supported other PCR data, showing that the transgene insertions were stably inherited via simple 3:1 Mendelian ratios in the chromosomal DNA. The two genes in event B2803 R6P1 were always inherited together and the plants used in the field trial were all homozygous.

### Analysis of *E*βf and other volatile compounds from transformed wheat plants

The emission levels of *E*βf from transformed plants in CE conditions varied with plant growth stage, rising until ~70 days post germination and subsequently declining. The same trend was observed in the field environment. A marked difference was observed between plants with the Eβ*fS* gene plastidially-targeted, which produced lower levels of *E*βf compared to plants with both the Eβ*fS* gene and *FPPS* gene plastidially-targeted, emission levels rising to a maximum of 3.8 and 30.7 μg/plant/h at 71 days post germination respectively (Fig. 1, Table S2, Fig. S4). No deleterious effects of the transformation on growth or development of plants were observed. The isoprenoid phytol, which relates biosynthetically to chlorophyll, and plant colour showed no difference between lines B2803 R6PI, B2812 R9PI and untransformed Cadenza. In addition to *E*βf, the monoterpene hydrocarbon myrcene, a compound with greater volatility than *E*βf, was detected at relatively higher ratios specifically in plants with only the gene encoding the plastidially-targeted Eβf synthase (Fig. 1, Table S2, Fig. S4). Coupled gas chromatography-electroantennography (GC-EAG) showed consistent electrophysiological responses to *E*βf in both constructs, with a lower response to myrcene (ratio of EAG signals, single construct plants: *E*βf 82.89% ± 1.68, myrcene 17.11% ± 1.68, 10 replicates).

### Aphid responses to transformed plants

**Figure 2:**
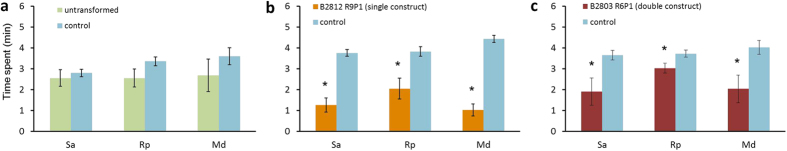
Time spent (mean ± s.e.) by cereal aphids *Sitobion avenae* (Sa), *Rhopalosiphum padi* (Rp) and *Metopolophium dirhodum* (Md) in different regions of a 4-arm olfactometer (n = 10). Aphids were exposed to four discrete odour streams, one treated with a headspace sample of volatiles collected from wheat seedlings, the other three treated with solvent (redistilled diethyl ether) control. Treatments that are significantly different (*P* < 0.05) are marked with an asterisk. (**a**) Aphids were not repelled by volatiles from untransformed Cadenza wheat seedlings. (**b,c**) All three aphid species spent significantly less time in the olfactometer region containing volatiles from the transformed wheat lines B2812 R9P1 and B2803 R6P1.

Three species of cereal aphid, economically important in the UK, were tested in the laboratory: grain aphid, *Sitobion avenae*, bird-cherry-oat aphid, *Rhopalosiphum padi*, and rose-grain aphid, *Metopolophium dirhodum*. Aphids were strongly repelled when exposed to volatiles collected from transformed plants grown under CE and field conditions. In an olfactometer bioassay, time spent in the area containing volatiles from transformed plants was compared with time spent in control areas. Aphids spent significantly less time in the treated area, indicating a repellent effect. Both the single (B2812 R9P1) and double construct (B2803 R6P1) lines were significantly repellent (Fig. 2, Fig. S5). The very high level of repellency for both lines did not allow for discrimination between the two at a behavioural level. Bioassays with myrcene indicated that it did not inhibit or otherwise influence the repellent response to *E*βf.

**Figure 3:**
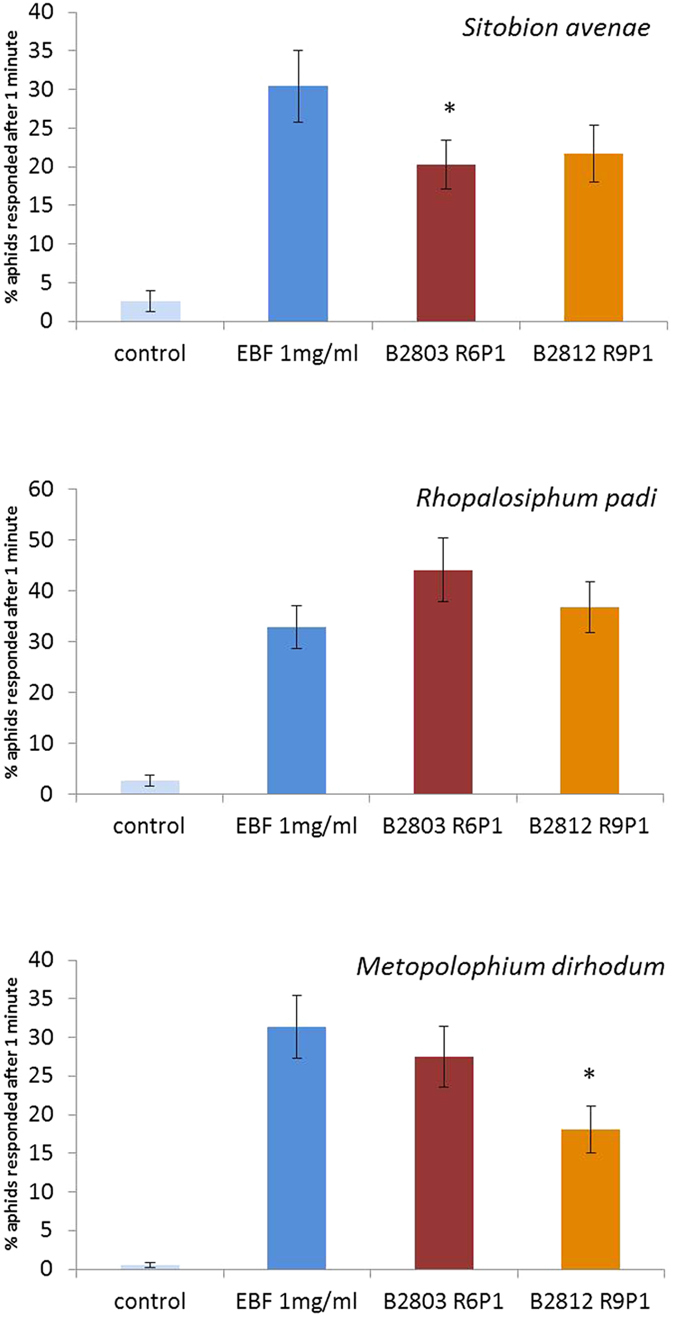
Alarm response of cereal aphid colonies (n = 17–19) to 1 μl drops of either samples of volatiles released by, B2812 R9P1 and B2803 R6P1 wheat plants containing 19 and 62 ng respectively, pure synthesised (*E*)-β-farnesene (1000 ng) or a solvent control: percentage of aphids that responded and/or moved away from the area after 1 minute (mean + /− s.e.). *indicates significant difference (*P* < 0.05) in response between treatment and (*E*)-β-farnesene.

Specific alarm responses of aphids to the transformed plants were also characterised using an alarm pheromone bioassay^3^, which measures disturbance of an aphid colony when exposed to sources of alarm pheromone. A strong alarm response was observed when aphid colonies on wheat leaves were exposed to volatiles from the transformed plants (Fig. 3). However, when aphids were reared on *E*βf emitting plants for five generations prior to testing, their responses in both the olfactometer and alarm pheromone bioassays were much reduced (Fig. S6). *R. padi* still exhibited an alarm pheromone response, but there was no significant response by *S. avenae*.

Aphid settlement on plants over a longer period was investigated in a field simulator trial. After a 24 h period, there was no significant difference in aphid settlement on transformed and untransformed plants for either *S. avenae* or *R. padi* (Fig. S7).

### Natural enemy responses to transformed plants

**Figure 4:**
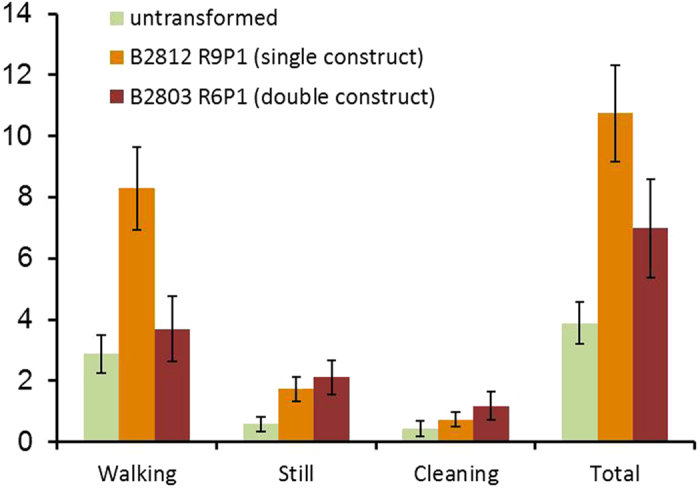
Time spent by foraging *Aphidius ervi* parasitoids on wheat seedlings (*n* = 15). Individual gravid females were released at the centre of the plant and time spent (mean ± s.e.) performing different behaviours was recorded until they left the plant. Compared to the untransformed wheat, total time spent was significantly longer on the transformed wheat lines B2812 R9P1 and B2803 R6P1 (*P* = 0.002).

A foraging bioassay, which examines the performance of parasitoids of aphids settled on plants, was used to compare the suitability of untransformed and transformed wheat plants for these key natural enemies of aphids. The total time spent foraging by the parasitoid *Aphidius ervi* was significantly longer on transformed plants (Fig. 4).

### Field trials of transformed wheat emitting Eβf

**Figure 5:**
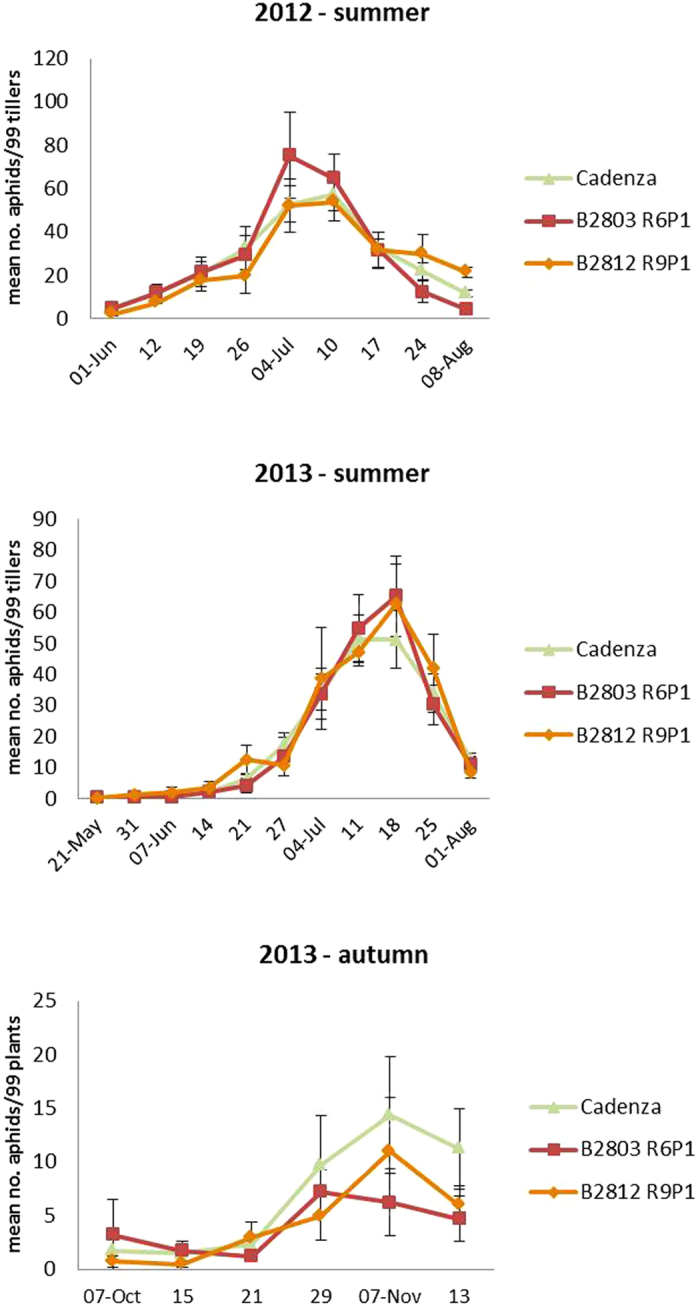
Aphid infestation levels in field plot trials. Ninety-nine plants or tillers were inspected in each plot each week. There were 4 plots of each of the transformed wheat lines B2812 R9P1 and B2803 R6P1 and eight plots of untransformed Cadenza wheat. No significant differences in infestation levels were detected.

Field trials were conducted on spring sown crops in 2012 and 2013 at Rothamsted, Harpenden, UK (map reference TL 120130), and on an autumn sown crop in 2013. This was intentionally terminated prior to flowering at the end of 2013 because it was designed to investigate effects on aphids specifically during the developmental stages of wheat when they transmit Barley Yellow Dwarf Virus. Two GM wheat lines, the single construct line (B2812 R9P1) and the double construct line (B2803 R6P1), were selected for field testing alongside the isogenic untransformed Cadenza variety used for transformation. Although the scope of these small-scale trials never included making commercially-relevant wheat yield measurements, we took samples and measured grain yields from each of the plots in the 2013 spring sowing. There was no statistically significant difference between the treatments. Aphid infestation levels in field plot trials were recorded on a weekly basis throughout the growing season in spring 2012, and in spring and autumn 2013. In both years, there was no significant difference in aphid populations between the plots with untransformed and transformed wheat (Fig. 5). The number of parasitized aphids was not different between treatments (Fig. S8).

## Discussion

The release of an insect pheromone, the aphid alarm pheromone (*E*βf), has been successfully achieved by genetic engineering of a crop plant, the hexaploid wheat cv. Cadenza. The pheromone released showed intrinsic activity against aphid pests and increased foraging by a natural enemy of aphids, the parasitic wasp *A. ervi,* in laboratory experiments. Habituation was observed in aphids reared on transformed plants. In field trials, the two levels of release of *E*βf achieved did not give rise to control of aphids or exploitation of natural enemies. Importantly, none of the experimental lines releasing Eβf showed any other noticeable phenotypic differences from the control lines.

Both aphid and parasitoid populations were low in the summer trials due to cold, wet weather, peaking at a mean of 0.76 aphids per tiller, with 20.33% of tillers infested, at the onset of anthesis on 4 July 2012 and 0.63 aphids per tiller, with 21.59% of tillers infested, at the end of anthesis on 18 July 2013. These are well below the recommended spray thresholds for summer aphids in the UK (50% of tillers infested before anthesis^13^). The plot size was small (6 m × 6 m), but has been successfully used in previous experiments showing efficacy of chemically derived alarm pheromone in the field^14^. Perhaps of relevance is the observation that, when aphids emit *E*βf after attack by a predator, this is via a sudden burst release from their siphunculi, followed by a rapid decline as the volatile compound dissipates and is oxidized. In contrast, our plants emitted *E*βf continuously and it is likely that this difference in the temporal release pattern meant that aphid settlement was not reduced in the field. Nonetheless, there should be an increase in parasitoid foraging behaviour, but perhaps only detectable at much higher populations, as can occur in some agroecological regions.

**Figure 6:**
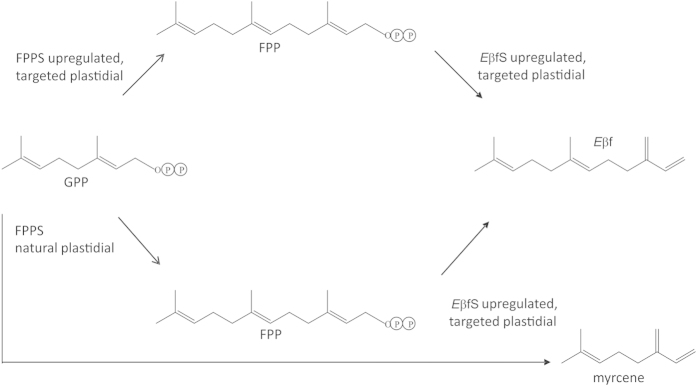
Scheme showing predominantly *E*βf from the double construct and the production of lower levels of myrcene from geranyl diphosphate (GPP) when the immediate precursor (FPP) is insufficiently available in the single construct (see Table S2).

The demonstration that plastidial targeting is necessary for satisfactory production of isoprenoids confirms, in a cereal crop, previous studies on the production of non-pheromonal isoprenoids^9^,^10^. Although sesquiterpenes can be naturally produced in the plastid^15^,^16^,^17^,^18^ (or via retargeting of biosynthetic enzymes), sesquiterpene synthases are normally localised in the cytosol^19^. In that respect, the failure to generate *E*βf as a result of the expression of cytosolic forms of *E*βfS and FPPS was unexpected and remains to be elucidated. In the case of plastidially-targeted *E*βfS in the absence of co-located FPPS, we suggest that, as a consequence of a high flux of the isoprenoid pathway in the plastid^20^ and because higher isoprenoids are produced, e.g. the carotenoids, there is a substantial, but transient, production of the precursor of sesquiterpenes, FPP. However, the access, by the sesquiterpene synthase enzyme in the transformed wheat, to this transient presence of FPP is limiting and so, for overexpression of the synthase *E*βfS without FPPS, the sesquiterpene synthase uses all natural FPP and then scavenges the homologous geranyl diphosphate, and thereby makes myrcene, the homologue of *E*βf, and at a relatively higher ratio than when the *E*βf precursor is more available (Fig. 6). This hypothesis also offers insights as to why, in *A. thaliana,* ubiquitous expression of *E*βfS without plastidial targeting could produce *E*βf and without myrcene, thus indicating greater access to FPP. Myrcene is often found in aphid host plants, but although associated with electrophysiological activity, as here, no role as a host plant cue is observed^21^.

This study is an important development in demonstrating, for the first time, stable genetic engineering of pheromone biosynthesis into crop plants. The work clearly shows that the concept needs to be tested further, with refinements as discussed above and more reliable natural populations of aphids and parasitoids. However, volatile semiochemicals produced on stress caused by herbivory, which repel herbivores and also attract beneficial insects, may also need to be considered as targets for genetic engineering.

## Methods

### Constructs and plant transformation. 

Chemically synthesised gene sequences, codon-optimised for wheat encoding enzymes [(*E*)-β-farnesene synthase (*E*βfS) and farnesyl diphosphate synthase (FPPS)], were produced by GenScript Inc. NJ, USA. The enzyme encoded by the Eβ*fS* cassette was similar to that found in peppermint (*Mentha piperita*) and the enzyme encoded by the *FPPS* cassette had most similarity to the form found in cow (*Bos taurus*). We chose this heterologous, synthetic gene to express FPP synthase to minimise the chances of co-suppressive silencing and to avoid any post-translational regulation by endogenous enzymes. Four constructs encoding *E*βfS or FPPS either targeted to the plastid or untargeted were made (*ubi*1::Eβ*fS*, *ubi*1:: tp:Eβ*fS*, *ubi*1::*FPPS*, *ubi*1:: tp:*FPPS* (Fig. S9). The targeting was achieved by incorporating a 147bp transit sequence from the small subunit of wheat RubisCo, previously validated to correctly target recombinant proteins to wheat plastids (Primavesi *et al.* 2008). To maximise our chances of successfully engineering the metabolic pathway to *E*βf, we chose to drive high levels of gene expression in a broad range of wheat tissues. Thus all constructs incorporated the maize *Ubi*1 promoter, previously shown to drive strong constitutive expression in wheat^9^. Genes were maintained on separate plasmid vectors based on the pGREEN2 plasmid (binary vector pBract309; www.bract.org) each containing a bar gene marker cassette for selection in tissue culture. Scutella of immature zygotic embryos isolated from the hexaploid wheat variety Cadenza (*Triticum aestivum,* Poaceae), were transformed by microprojectile bombardment^22^. Whole plants were regenerated and selected from somatic embryos induced in tissue culture on medium containing the herbicide glufosinate ammonium. Healthy seedlings were transferred to soil to continue growth to maturity. The presence of the transgene(s) was confirmed by PCR and expression of functional enzymes implied from measuring an altered plant volatile profile. Copy number was determined by quantitative (TaqMan) PCR performed on genomic DNA isolated from several T1 plants from each event by iDNA Genetics (Norwich, UK), using regions of the coding sequence as primers/probes.

### Air entrainment and GC-MS

All plants were grown and maintained in a specialised, air conditioned glass house under controlled conditions (16 h:8 h light:dark regime; 20 °C:15 °C day:night temperatures). To determine whether transgenic wheat lines were emitting *E*βf, the headspace above leaves was sampled as described previously^23^. Plants at various stages of growth after sowing (Table S2), were enclosed in a glass vessel, the size of which depended on the size of the plant, or a polyethylene terephthalate (PET) oven bag (250 mm × 380 mm) for air entrainment^24^. Volatiles were collected on Porapak Q (50 mg, 60/80 mesh; Supelco, Bellefonte, PA, USA) absorbent tubes inserted into the collection ports on top of the vessels. Porapak Q filters were eluted with 0.75 ml of redistilled diethyl ether after a 24 h collection period. Headspace samples were analysed by GC-MS. A 2 μl aliquot of the air headspace sample was injected onto a capillary GC (Agilent 6890) directly coupled to a mass spectrometer (MS) (Agilent 5973 MSD). Ionization was achieved by electron impact at 70 eV, 250 °C. The oven temperature was maintained at 30 °C for 5 min and then programmed at 5 °C min^−1^ to 250 °C. Identification of *E*βf, myrcene and phytol was confirmed by comparison of mass spectra and co-injection with an authentic standards (*E*βf, > 98% purity, synthesised at Rothamsted) on two columns of different polarity (non-polar, HP-1 column, 50 m, 0.32 mm i.d., 0.52 lm film thickness; polar DB-wax column, 30 m, 0.32 mm i.d., 0.5 lm film thickness). Quantification was carried out by calculating and comparing peak areas with known amounts of authentic external standards. Two transformed lines, B2812 R9P1 (single construct) and B2803 R6P1 (double construct), were chosen for air entrainment on the basis of *E*βf emission and low copy number. Volatiles were collected from 5 week old seedlings to provide the headspace samples used in bioassays. These lines were also used to bulk up seed for field trials. In additional entrainments, volatiles were sampled from transformed plants in the T2 and T3 generations at different time points to investigate emission at different ages and fresh weight was recorded (see data for T3 in Table S2).

### Electrophysiology

Electroantennogram (EAG) recordings were made using Ag-AgCl glass electrodes filled with saline solution (composition as in Maddrell, 1969^25^, but without glucose). The head of an alate aphid was excised and placed within the indifferent electrode, with the tips of the antennae being inserted into the recording electrode. The signals were passed through a high impedance amplifier (UN-06, Syntech, The Netherlands) and analysed using a customized software package (Syntech). The coupled GC-electrophysiology system, in which the effluent from the GC column is simultaneously directed to the antennal preparation and the GC detector, has been described previously^26^. Separation of the volatiles was achieved on a GC (Agilent Technologies, 6890N) equipped with a cold on-column injector and an FID. For the HP-1 column, the oven temperature was maintained at 30 °C for 2 minutes and then programmed at 15° min^−1^ to 250 °C. The carrier gas was helium. The outputs from the EAG amplifier and the FID were monitored simultaneously and analysed using the Syntech software package. GC peaks were judged to be active if EAG responses were observed in three or more coupled runs. Responses were obtained from volatiles entrained from the headspace above *E*βfS-expressing wheat B2812 R9P1 or B2803 R6P1 plants, 71 days post sowing, releasing 3.82 and 30.7 μg/plant/h respectively.

### Aphid alarm response bioassay

Experimental procedure was similar to that used in previous studies^6^,^12^. Replicated colonies (5–7/treatment/trial) of 20–40 individual aphids (*S. avenae*, *M. dirhodum* or *R. padi*) feeding on cut leaves of wheat (cv. Cadenza, or appropriate GM line when testing the response of aphids reared on transformed lines for 5 generations) were placed in a constant flow of air. A 1 μl droplet of diethyl ether (or hexane for the assays with aphids reared on transformed lines for 5 generations), containing either 98% pure synthetic *E*βf (1000 ng), or volatiles entrained from the headspace above *E*βfS-expressing wheat (B2812 R9P1 and B2803 R6P1, 35 days post sowing, containing 19 and 62 ng *E*βf μl^−1^ respectively) or untransformed plants, was applied to the leaf and the insect behavior observed (Fig. 3, Fig. S6). Individual treatments were randomized within each trial. Depending on the target species, the numbers of aphids responding and/or moving off the leaf were counted after 1 min. Data were analysed by ANOVA (Genstat 15^th^ edition) after arcsin transformation.

### Aphid olfactometer bioassay

Individual aphids (*S. avenae*, *M. dirhodum* or *R. padi*) were introduced via the central exit port into a four-arm olfactometer^12^, which provided a 10 cm diam. star shaped-arena for aphids to walk in. Air was drawn (260 ml min^−1^) through four arms to exit at the centre. A 10 μl headspace sample from either a transgenic *E*βf-producing line (B2812 R9P1 and B2803 R6P1) or from an untransformed plant was placed on a filter paper in the appropriate side, through which air was drawn into the part of the arena receiving treatment. Control areas received air passing over solvent (10 μl diethyl ether) on filter paper. The length of time spent in the treated part of the arena was recorded and compared to the control part of the arena. Individual aphids were observed over a 16 minute period in each replicate of the bioassay and data analysed by t-test (Genstat 16^th^ edition).

### Parasitoid foraging bioassay

Experimental procedure was similar to previous work^3^ and was conducted with *Aphidius ervi*. Individual 2-week old wheat seedlings (cv. Cadenza, B2812 R9P1 or B2803 R6P1) were mounted on a turntable which allowed them to be examined even when the parasitoid moved to the back of the plant. Once an individual parasitoid was released, the time it spent walking, remaining still or cleaning itself was recorded. An observation was terminated when a parasitoid flew away from the plant. Noldus Observer 5 software was used for recording the behavioral observations.

### Field simulator

Cadenza, B2812 R9P1 or B2803 R6P1 plants were tested under no-choice conditions in a Perspex simulator (90 × 30 × 30 cm, wind speed 0.14 m s^−1^, 22 °C, 40% RH). Sixteen, 7-day old seedlings, sown in a grid of 2 × 2 cm square pots, were positioned at the upwind end of the simulator. One hundred alate *S. avenae* or *R. padi*, which had acclimatized to the room conditions for 1 h prior to the start of the experiment, were released into a Petri dish lined with moistened filter paper at the downwind end. Counts of settled aphids were made 2, 5 and 24 h after release, and settlement on treated seedlings was compared with settlement on control seedlings using an unpaired *t*-test (Genstat 16^th^ Edition).

### Field trials

Permission for the trials was granted in Sept 2011 by Defra (Department of Environment, Food and Rural Affairs) after an assessment of the risks to human health and the environment, a period of public consultation and advice from ACRE (the Advisory Committee on Releases in the Environment).

The trial to evaluate levels of aphid infestation and parasitism was conducted on Rothamsted farm. A 4 × 4 Latin square design of 6 m × 6 m plots was used, similar to previous semiochemical trials^14^. Treatments comprised two transformed wheat lines, B2812 R9P1 (single construct) and B2803 R6P1 (double construct) (4 plots each), plus a control treatment of untransformed Cadenza (8 plots). Plots were separated from each other by 10 m (0.5 m space, 9 m barley, 0.5 m space) and from the edge of the trial by 7 m of barley (0.5 m space, 6 m barley, 0.5 m space), plus an outer 6 m pollen barrier of wheat (Cadenza). Two 2.4 m high chain-link fences surrounded the trial to prevent the entry of rabbits and unauthorised persons. Spring trials were sown on 22 March 2012, and 10 April 2013 and the autumn trial on 24 September 2013. Inspections of cereal aphid (*S. avenae*, *M. dirhodum* and *R. padi*) infestation were made weekly from May to August in the spring sown trials, and in October and November for the autumn sowing. The central 5 × 5 m of each plot was marked with a 3 × 3 grid of stakes 2.5 m apart. Eleven plants/tillers were inspected at each stake on each sampling occasion (i.e. 99 plants per plot). Numbers of predators, parasitoids and parasitized aphid mummies were also recorded. Transformed data [y = log (y + 1)] were analysed by ANOVA.

## Additional Information

**How to cite this article**: Bruce, T.J.A. *et al.* The first crop plant genetically engineered to release an insect pheromone for defence. *Sci. Rep.* **5**, 11183; doi: 10.1038/srep11183 (2015).

## Supplementary Material

Supplementary Information
