# Supplementary material for: The first crop plant genetically engineered to release an insect pheromone for defence
Source: Sci Rep. 2015 Jun 25;5:11183. doi: 10.1038/srep11183 (PMC4480004; doi:10.1038/srep11183)

**The first crop plant genetically engineered to release an insect pheromone for defence**

Toby J.A. Bruce, Gudbjorg I. Aradottir, Lesley E. Smart, Janet L. Martin, John C. Caulfield, Angela Doherty, Caroline A. Sparks, Christine M. Woodcock, Michael A. Birkett, Johnathan A. Napier, Huw D. Jones and John A. Pickett

Rothamsted Research, Harpenden, AL5 2JQ, UK

Corresponding author: John A. Pickett (john.pickett@rothamsted.ac.uk)

SUPPLEMENTARY FIGURE LEGENDS

**Table S1**  TaqMan analysis of copy number for events B2803 R6P1 and B2812 R9P1. Genomic DNA from segregating T1 plants from each event was subjected to TaqMan analysis for Eβ*fS* and *FPPS* as appropriate (for B2803 R6P1 both gene targets were used, for B2812 R9P1 only Eβ*fS* was used). For B2803 R6P1, 17 randomly selected T1 plants were analysed in duplicate. For B2812 R9P1, 15 T1 plants already known to possess the EBF cassette were analysed in a single assay run.

| Sample | Eβ*fS* Copies | Call | *FPPS* Copies | Call | Comp. plates 1 & 2 |
| --- | --- | --- | --- | --- | --- |
| 803R6P1-1_1 | 2 | Hom | 2 | Hom | Match |
| 2803R6P1-1_2 | 2 | Hom | 2 | Hom |  |
| 2803R6P1-12_1 | 2 | Hom | 2 | Hom | Match |
| 2803R6P1-12_2 | 2 | Hom | 2 | Hom |  |
| 2803R6P1-14_1 | 2 | Hom | 2 | Hom | Match |
| 2803R6P1-14_2 | 2 | Hom | 2 | Hom |  |
| 2803R6P1-5_1 | 2 | Hom | 2 | Hom | Match |
| 2803R6P1-5_2 | 2 | Hom | 2 | Hom |  |
| 2803R6P1-9_1 | 2 | Hom | 2 | Hom | Match |
| 2803R6P1-9_2 | 2 | Hom | 2 | Hom |  |
| 2803R6P1-10_1 | 1 | Hemi | 1 | Hemi | Match |
| 2803R6P1-10_2 | 1 | Hemi | 1 | Hemi |  |
| 2803R6P1-11_1 | 1 | Hemi | 1 | Hemi | Match |
| 2803R6P1-11_2 | 1 | Hemi | 1 | Hemi |  |
| 2803R6P1-16_1 | 1 | Hemi | 1 | Hemi | Match |
| 2803R6P1-16_2 | 1 | Hemi | 1 | Hemi |  |
| 2803R6P1-17_1 | 1 | Hemi | 1 | Hemi | Match |
| 2803R6P1-17_2 | 1 | Hemi | 1 | Hemi |  |
| 2803R6P1-2_1 | 1 | Hemi | 1 | Hemi | Match |
| 2803R6P1-2_2 | 1 | Hemi | 1 | Hemi |  |
| 2803R6P1-4_1 | 1 | Hemi | 1 | Hemi | Match |
| 2803R6P1-4_2 | 1 | Hemi | 1 | Hemi |  |
| 2803R6P1-6_1 | 1 | Hemi | 1 | Hemi | Match |
| 2803R6P1-6_2 | 1 | Hemi | 1 | Hemi |  |
| 2803R6P1-7_1 | 1 | Hemi | 1 | Hemi | Match |
| 2803R6P1-7_2 | 1 | Hemi | 1 | Hemi |  |
| 2803R6P1-8_1 | 1 | Hemi | 1 | Hemi | Match |
| 2803R6P1-8_2 | 1 | Hemi | 1 | Hemi |  |
| 2803R6P1-13_1 | 0 | Null | 0.1 | <1 | Match |
| 2803R6P1-13_2 | 0 | Null | 0.1 | <1 |  |
| 2803R6P1-15_1 | 0 | Null | 0 | Null | Match |
| 2803R6P1-15_2 | 0 | Null | 0 | Null |  |
| 2803R6P1-3_1 | 0 | Null | 0 | Null | Match |
| 2803R6P1-3_2 | 0 | Null | 0 | Null |  |

| Sample | Eβ*fS*copies | Call |
| --- | --- | --- |
| 2812R9P1_13 | 8 | Hom |
| 2812R9P1_14 | 8 | Hom |
| 2812R9P1_4 | 8 | Hom |
| 2812R9P1_1 | 4 | Hemi |
| 2812R9P1_10 | 4 | Hemi |
| 2812R9P1_11 | 4 | Hemi |
| 2812R9P1_12 | 4 | Hemi |
| 2812R9P1_15 | 4 | Hemi |
| 2812R9P1_2 | 4 | Hemi |
| 2812R9P1_3 | 4 | Hemi |
| 2812R9P1_5 | 4 | Hemi |
| 2812R9P1_6 | 4 | Hemi |
| 2812R9P1_7 | 4 | Hemi |
| 2812R9P1_8 | 4 | Hemi |
| 2812R9P1_9 | 4 | Hemi |

**Figure S1** Gas chromatography (GC) analysis of volatiles released from transformed wheat plants without plastidial targeting: (upper trace) plant possessing gene encoding *E*βf synthase alone (lower trace) plant possessing genes encoding *E*βf synthase and FPP synthase . Arrow = retention time at which (*E*)- β-farnesene (*E*βf) would elute, 2 = BHT (antioxidant contaminant)

**
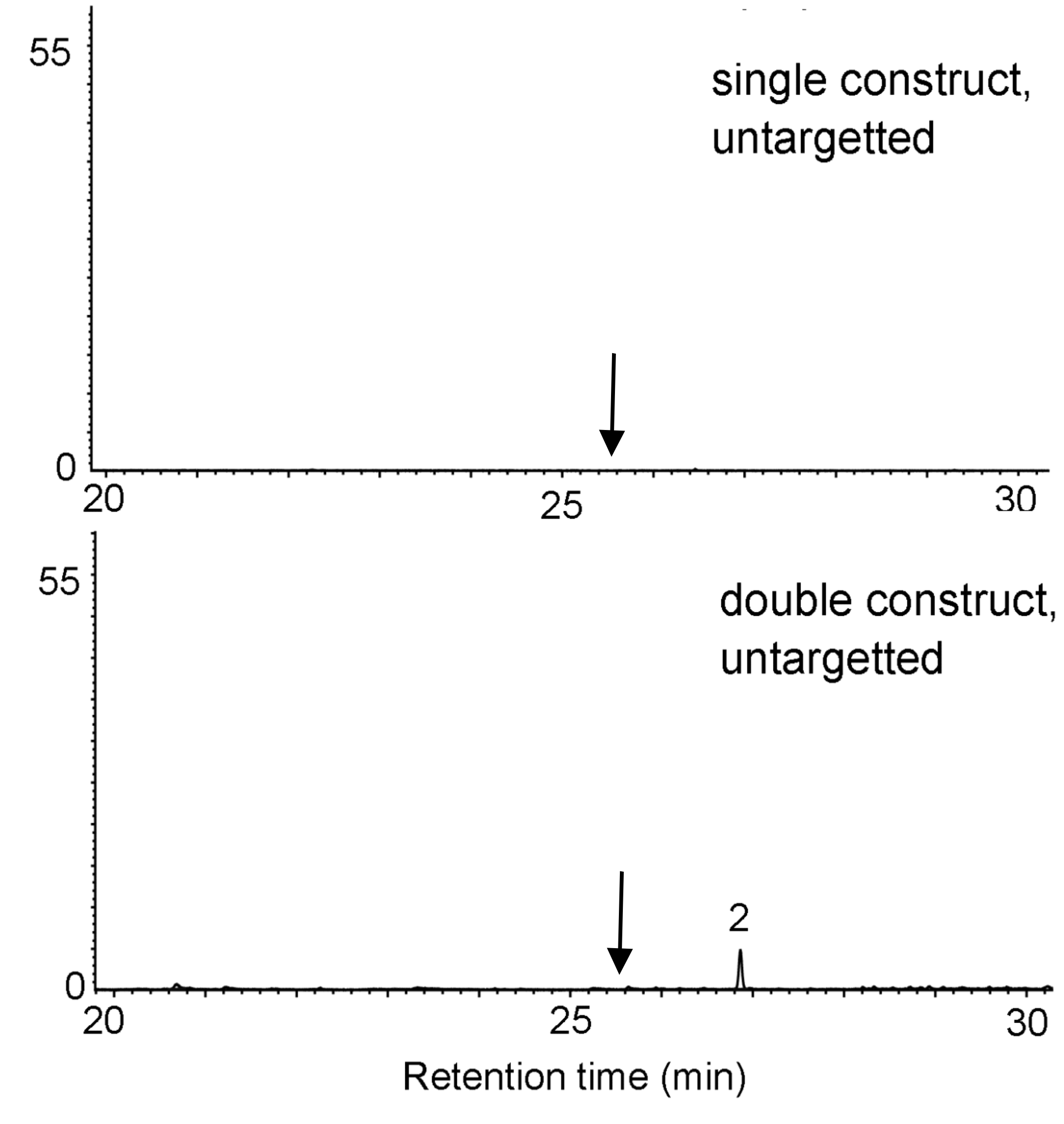
**

**Figure S2**

**
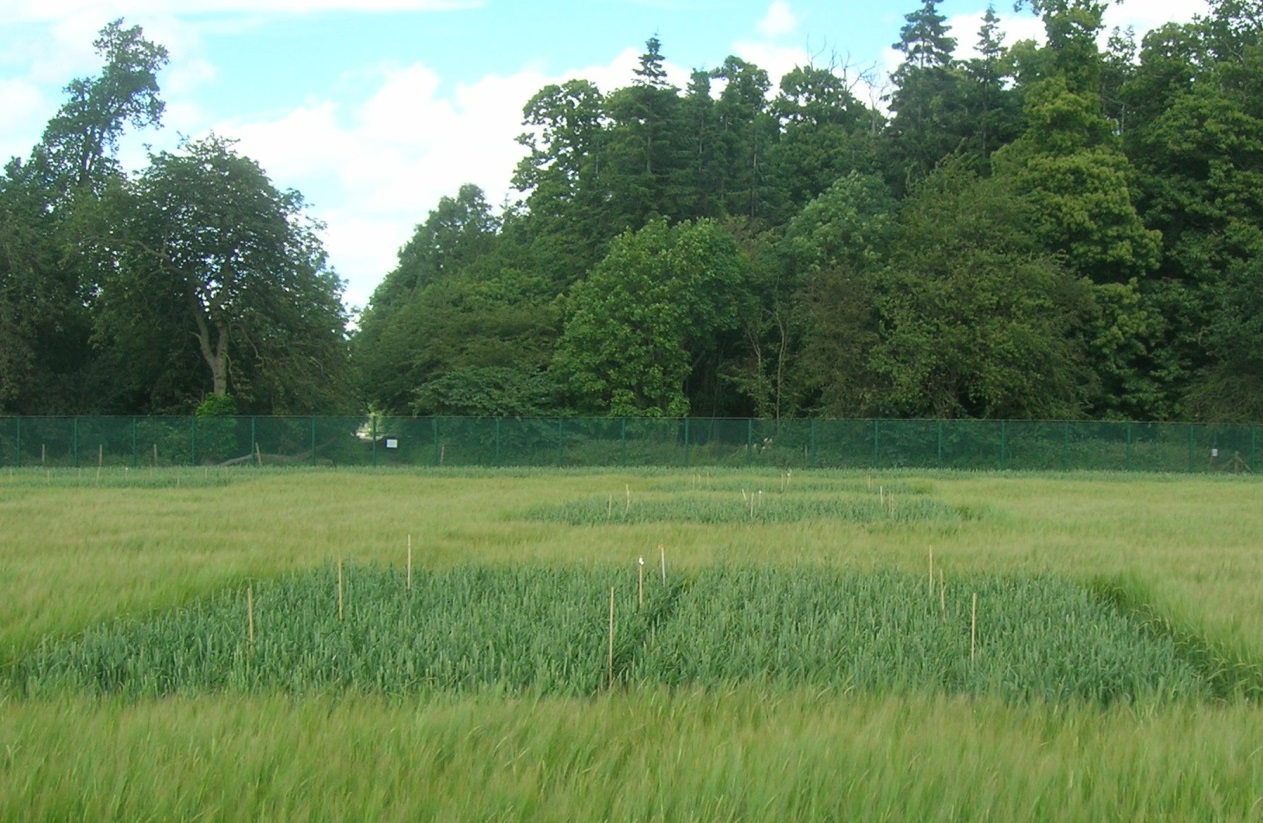
**


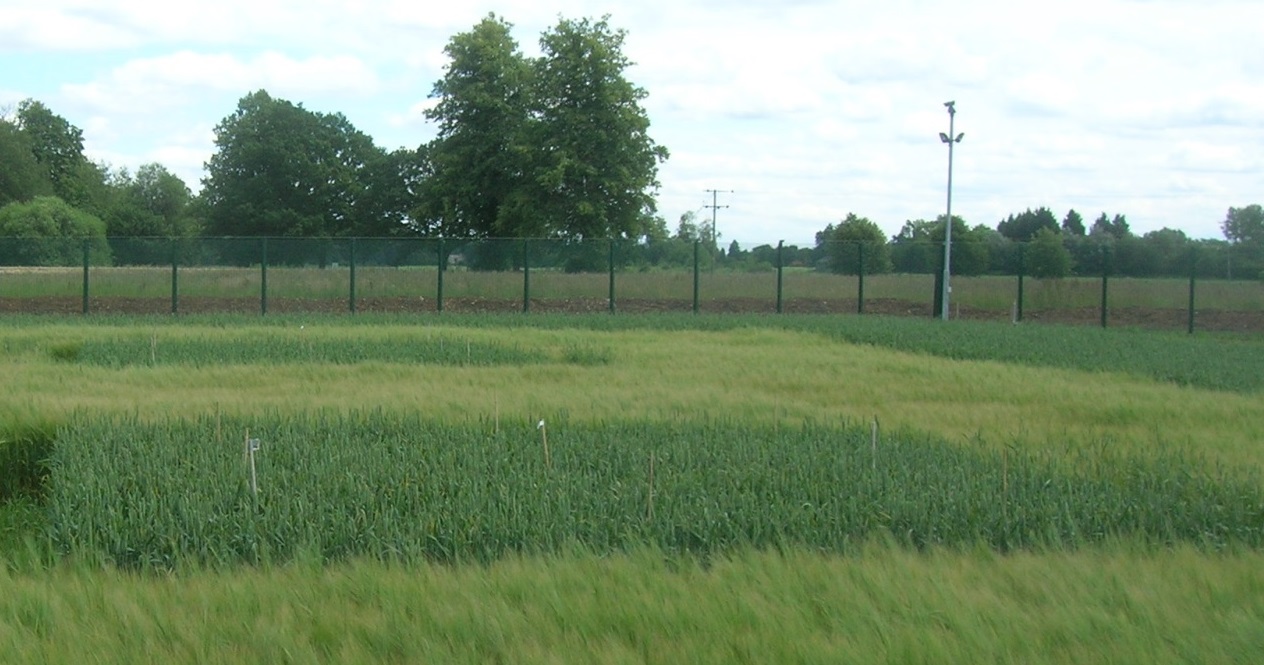


B2812 R9P1

(plot 7)

Control

(plot 11)

Control

(plot 2)

B2812 R9P1

(plot 1)

Photos taken 11.7.12 by Huw D. Jones


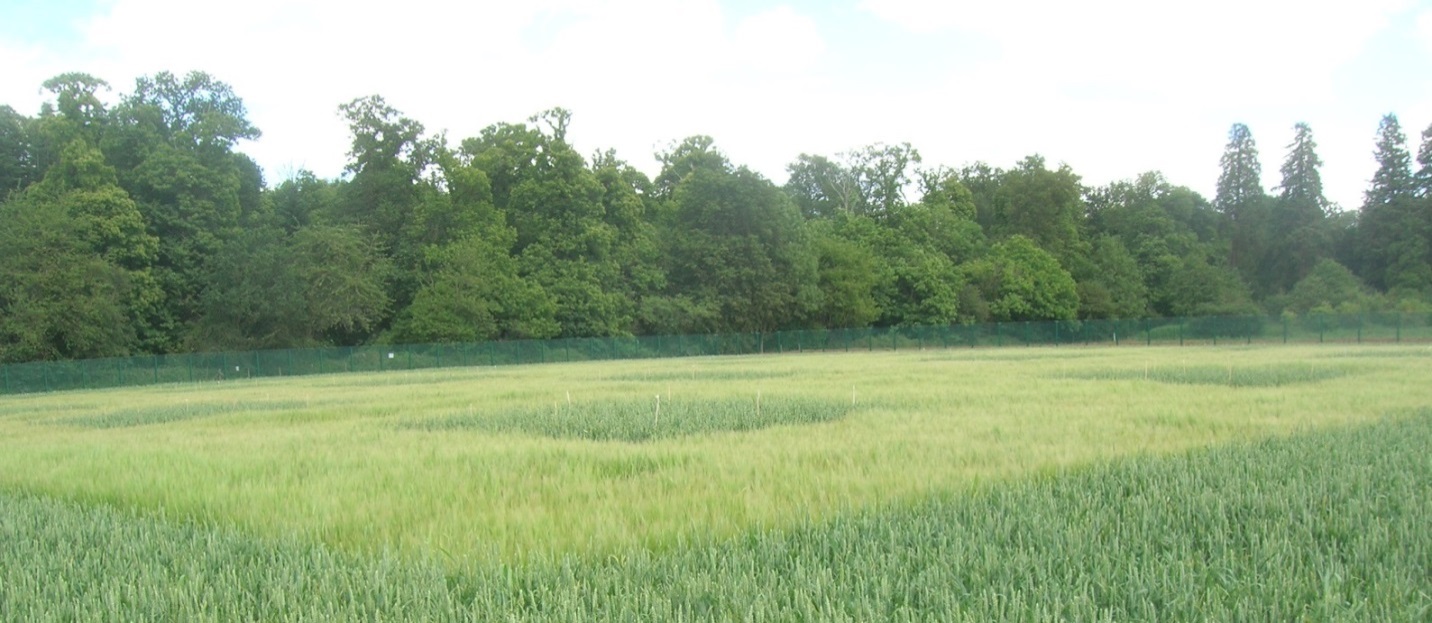


Control

(plot 4)

B2803 R6P1

(plot 3)

B2803 R6P1

(plot 8)

B2812 R9P1

(plot 7)

Photo taken 11.7.12 by Huw D. Jones

**Figure S3** SPAD readings and photographs taken from leaves of control and GM plants. Readings were taken with **a** Minolta chlorophyll meter (SPAD-502) and show output and means from two leaves of plant 1 (23 days old) and one leaf each of three younger plants (15 days old). Four or five readings were taken according to the leaf size from the base to the tip of the leaf.

|  | **Control Cadenza** | | | | | **Line B2812 R9P1 (single construct)** | | | | | **Line B2803 R6P1 (double construct)** | | | | |
| --- | --- | --- | --- | --- | --- | --- | --- | --- | --- | --- | --- | --- | --- | --- | --- |
|  | Plant 1 | | Plant 2 | Plant 3 | Plant 4 | Plant 1 | | Plant 2 | Plant 3 | Plant 4 | Plant 1 | | Plant 2 | Plant 3 | Plant 4 |
|  | Leaf 1 | Leaf 2 |  |  |  | Leaf 1 | Leaf 2 |  |  |  | Leaf 1 | Leaf 2 |  |  |  |
| Tip of leaf | 44.4 | 47.0 | 41.0 | 34.4 | 34.9 | 44.2 | 42.4 | 38.6 | 42.5 | 43.0 | 44.5 | 44.3 | 38.0 | 38.2 | 46.0 |
|  | 42.5 | 42.1 | 38.3 | 36.8 | 37.1 | 40.3 | 46.7 | 34.9 | 37.9 | 36.5 | 42.7 | 47.9 | 35.4 | 36.6 | 43.3 |
|  | 34.9 | 43.1 | 34.9 | 37.8 | 32.2 | 38.1 | 43.4 | 32.8 | 38.7 | 34.4 | 38.5 | 44.4 | 31.9 | 40.5 | 33.4 |
|  | 31.0 | 37.4 | 29.6 | 31.8 | 28.1 | 31.7 | 37.8 | 26.1 | 28.6 | 25.3 | 30.3 | 40.9 | 26.9 | 34.1 | 28.2 |
| Base of leaf | 26.8 | N/A | N/A | N/A | N/A | 28.6 | N/A | N/A | N/A | N/A | 29.9 | N/A | N/A | N/A | N/A |
| Mean | 35.9 | 42.4 | 36.1 | 35.2 | 33.1 | 36.6 | 42.6 | 33.1 | 36.9 | 34.8 | 37.2 | 44.4 | 33.1 | 37.4 | 37.7 |
| Overall mean | 36.54 | | | | | 36.8 | | | | | 37.96 | | | | |


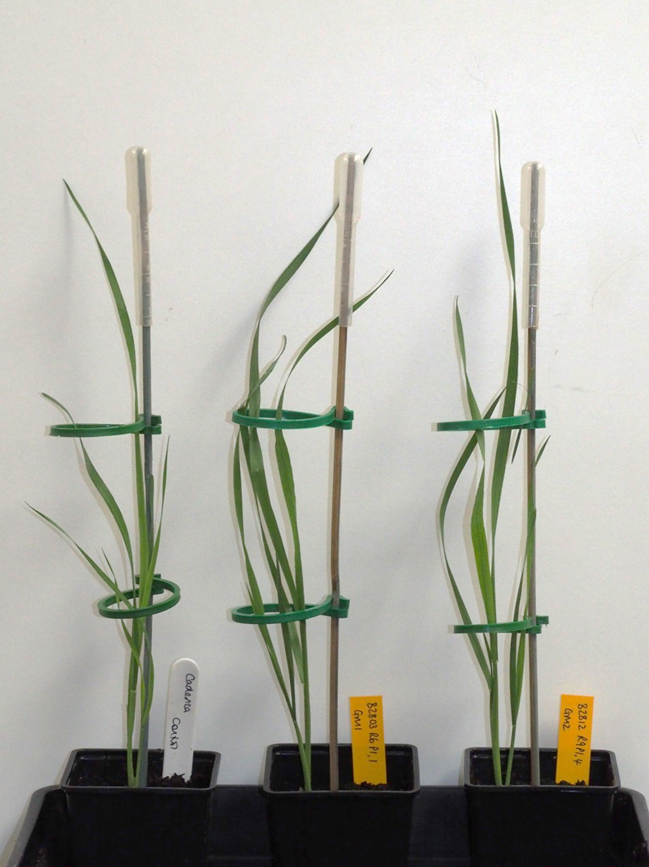


23 days old (Control, B2803 R6P1, B2812 R9P1).


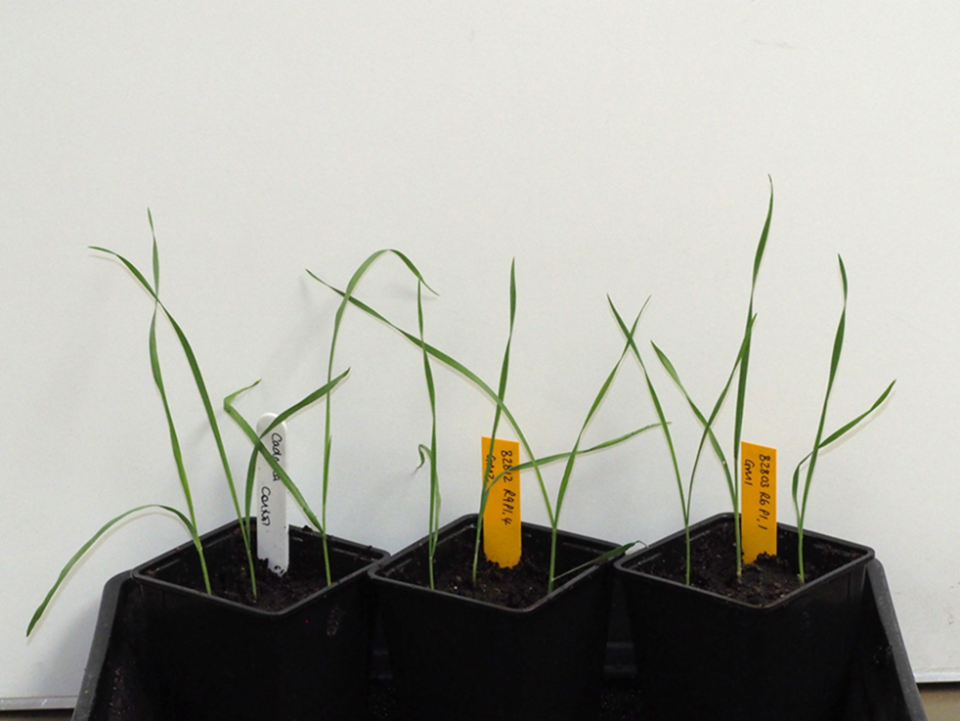


15 days old (Control, B2812 R9P1, B2803 R6P1). All photos in this figure were taken by Caroline A. Sparks.

**Figure S4** Volatile emissions of *E*βf and myrcene from transformed T3 wheat plants presented as µg/plant/h: (upper trace) B2812 R9P1 plastidially-targeted *E*βf synthase alone, (lower trace) B2803 R6P1 *E*βf synthase + FPP synthase, both with plastidial targeting sequence.


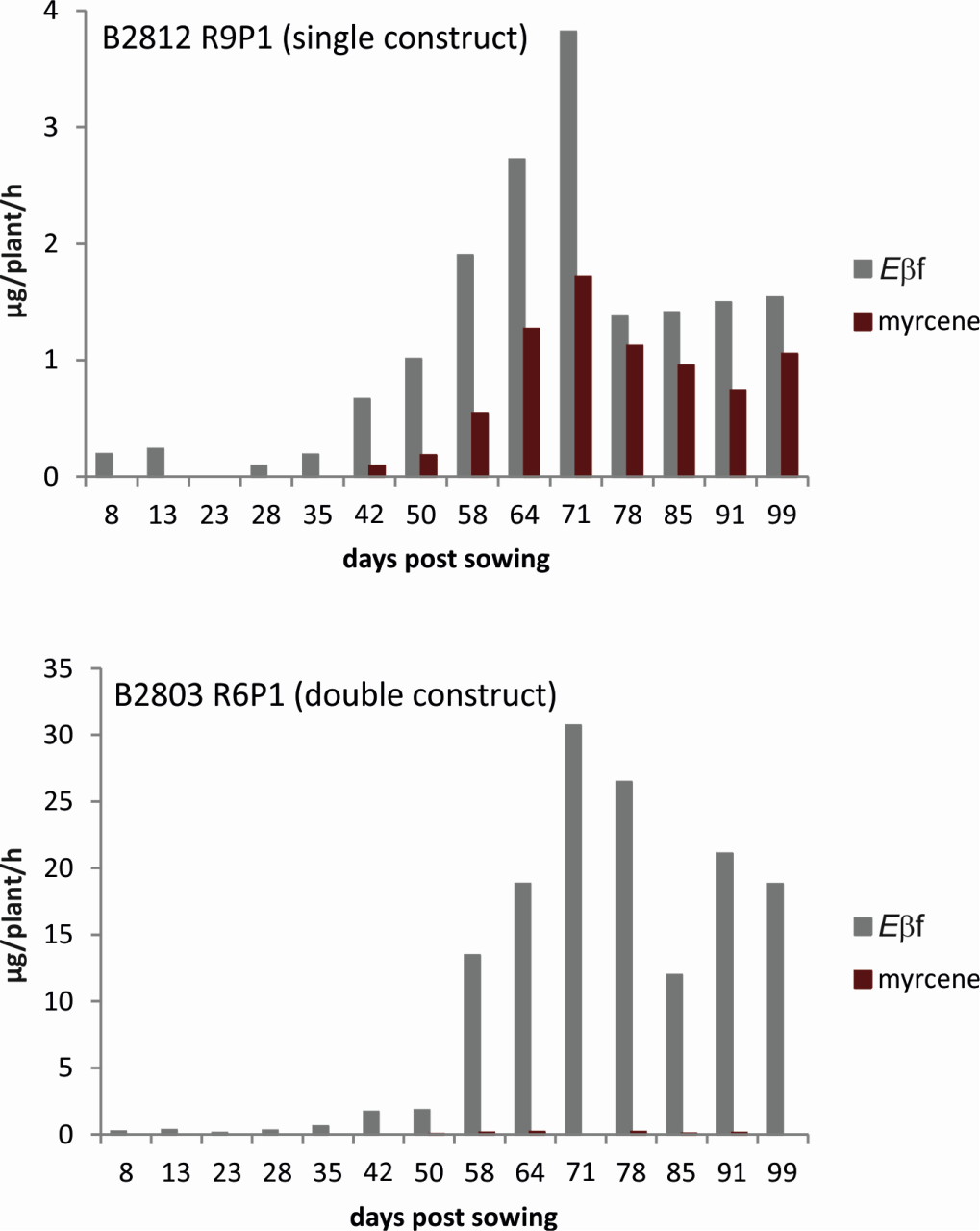


**Figure S5** Time spent (mean ± s.e.) by cereal aphids, *Sitobion avenae* and *Rhopalosiphum padi* in different regions of a 4-arm olfactometer (*n* = 10). Aphids were exposed to four discrete odour streams, one treated with a headspace sample of volatiles collected from field grown transformed wheat lines B2812 R9P1 and B2803 R6P1, the other three treated with solvent (redistilled diethyl ether) control. Treatments that are significantly different (*P* < 0.05) are marked with an asterisk.


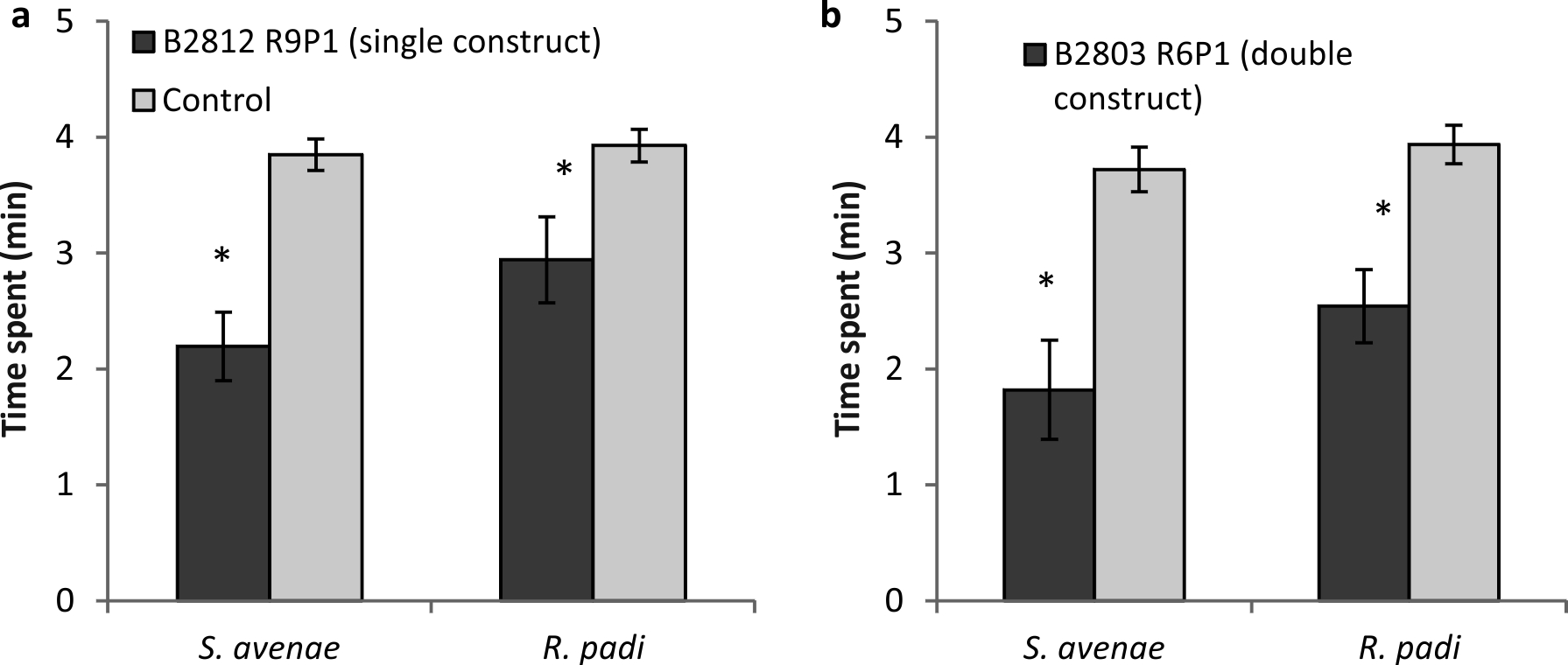


**Figure S6** Alarm pheromone response: percentage of aphids that responded and/or moved away from the area after 1 minute (mean +/- s.e.). and olfactometer response: time spent in different regions of a 4-arm olfactometer (mean ± s.e.) of aphids, reared for 5 generations on Cadenza or transformed wheat lines B2812 R9P1 or B2803 R6P1, to pure synthesised (*E*)-β-farnesene (1000ng) or a solvent control.

**
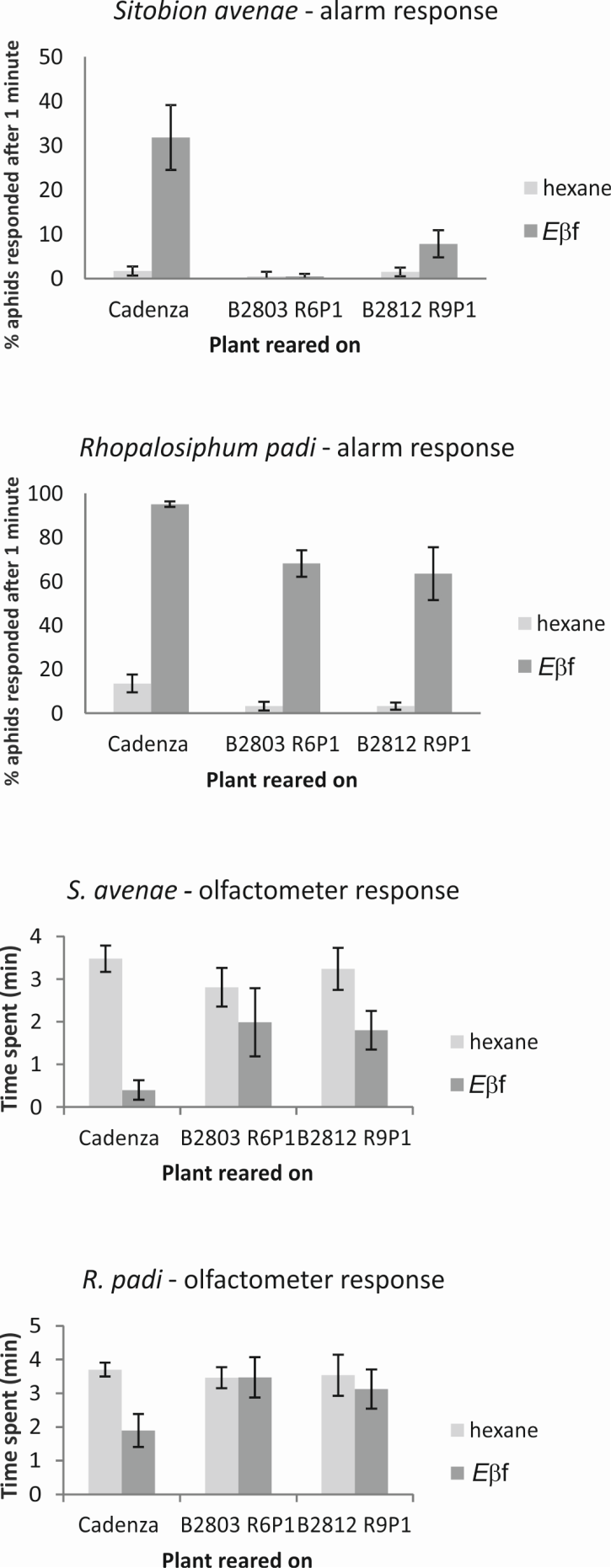
**

**Figure S7** Aphid settlement in a field simulator. One hundred winged aphids were released in each replicate and the number settled on 16, 7 day old seedlings of Cadenza, B2803 R6P1 or B2812 R9P1 was recorded after 2 h, 5 h and 24 h. No significant differences in infestation levels were detected.


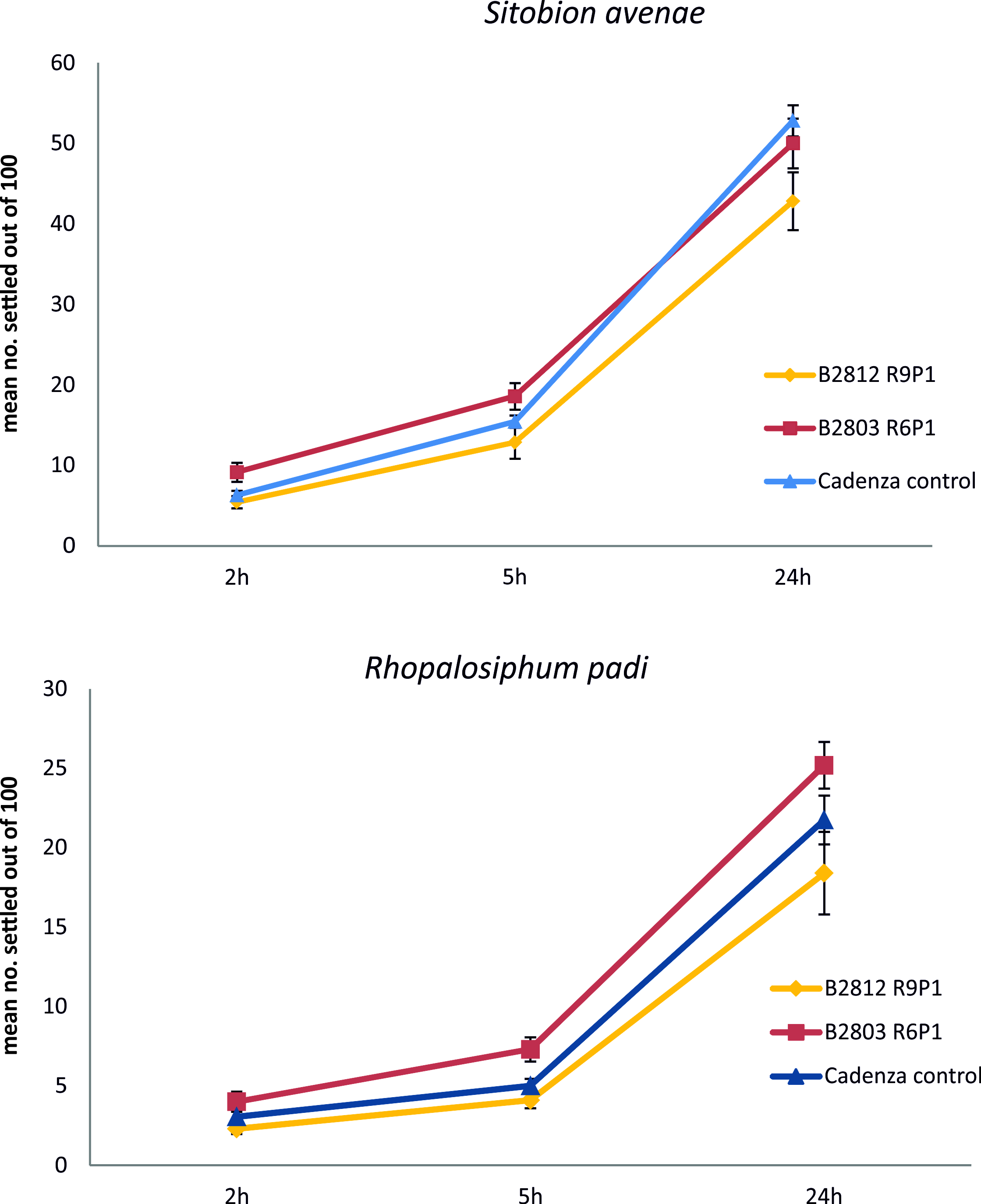


**Figure S8** Aphid parasitism levels in the field trials represented as the mean number of parasitized aphids on wheat plants. No significant differences in parasitism levels were detected.

**
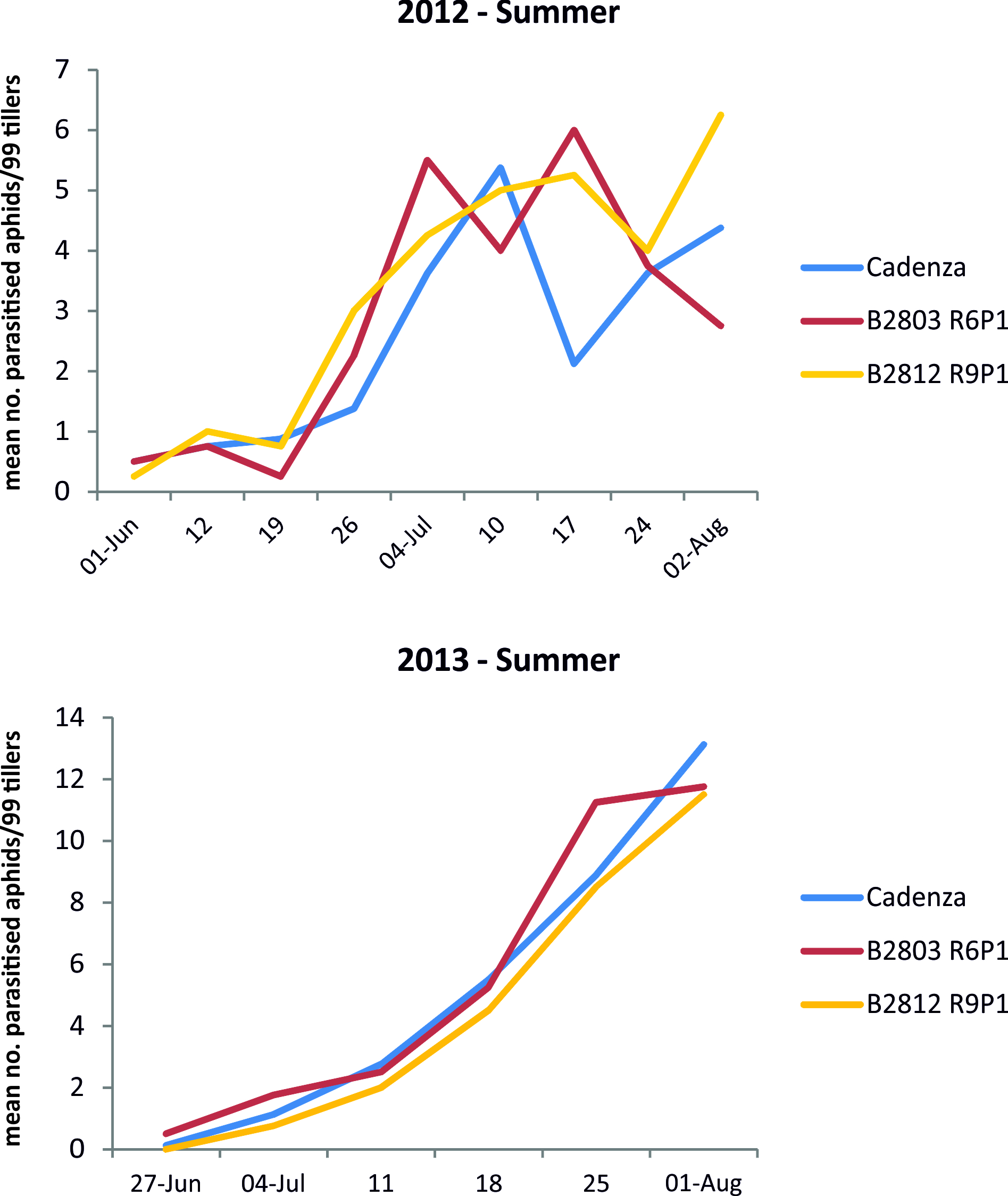
**

**Figure S9** Transformation vectors were constructed with Eβ*f* synthase or *FPPS* synthase expression cassettes, either untargeted or targeted to the plastid, using a 147bp transit sequence from the small subunit of RubisCo. The two targeted forms are shown here (*ubi*1:: *tp*:Eβ*fS*, *and*  *ubi*1:: *tp:FPPS*). Genes were maintained on separate plasmid vectors based on the pGREEN2 plasmid (binary vector pBract309; [www.bract.org](http://www.bract.org/)) each containing a bar gene marker cassette for selection in tissue culture.


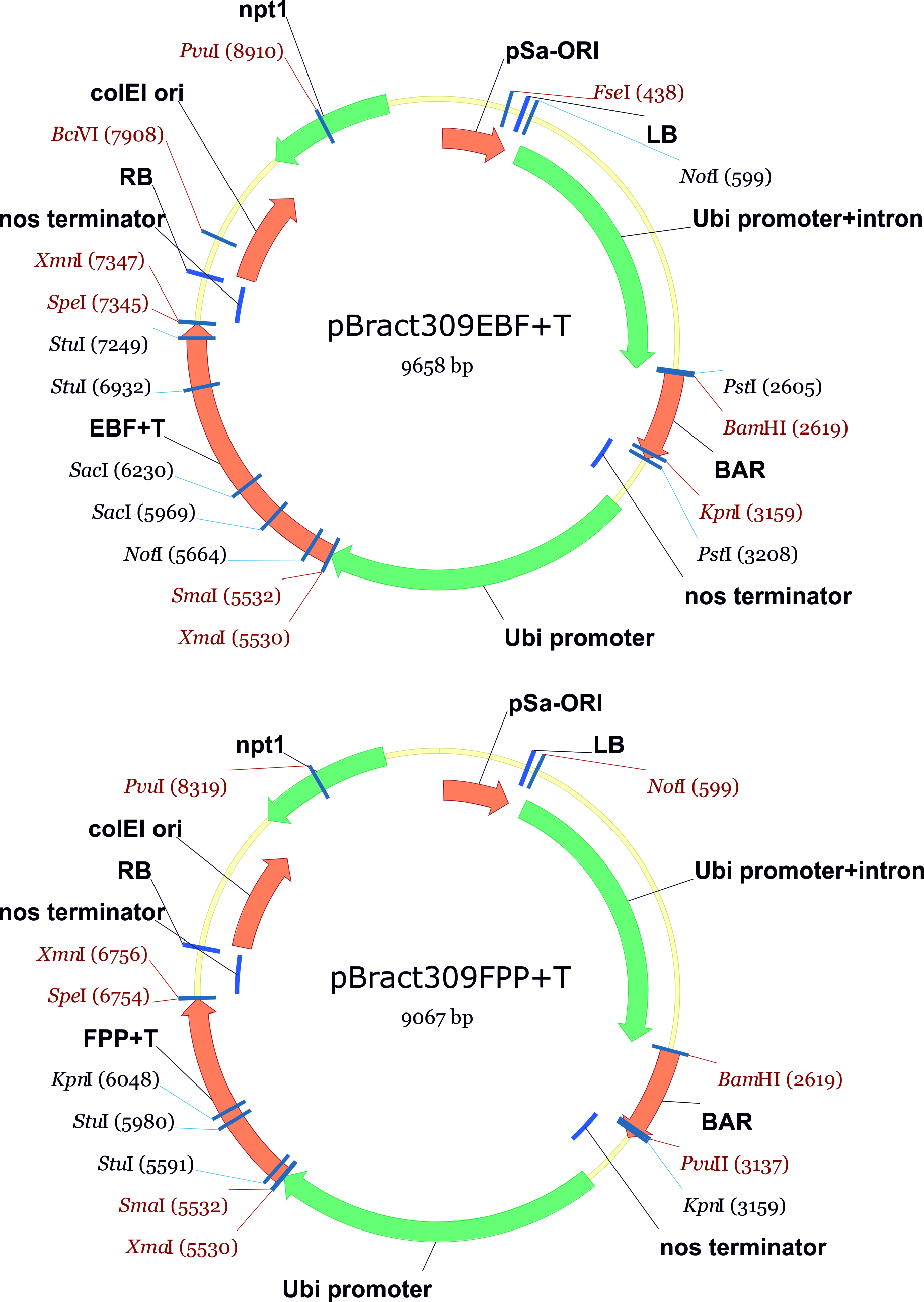

Supplement: Supplementary Information [file srep11183-s1.doc]
